# Repeat sprint fatigue and altered neuromuscular performance in recreationally trained basketball players

**DOI:** 10.1371/journal.pone.0288736

**Published:** 2023-07-17

**Authors:** Nicolas M. Philipp, Dimitrije Cabarkapa, Drake A. Eserhaut, Daniel Yu, Andrew C. Fry

**Affiliations:** 1 Jayhawk Athletic Performance Laboratory, Wu Tsai Human Performance Alliance – University of Kansas, University of Kansas, Lawrence, Kansas, United States of America; 2 Orlando Magic, Orlando, Florida, United States of America; Instituto Politécnico de Santarém: Instituto Politecnico de Santarem, PORTUGAL

## Abstract

The primary aim of the present study was to investigate how the fatigue induced through a repeat sprint protocol acutely affected different measures of neuromuscular performance. Recreationally trained basketball players (n = 25) volunteered to participate in the study, and performed three countermovement jumps (CMJ), as well as three drop jumps (DJ) prior to a fatiguing repeat sprint protocol. These procedures were repeated two minutes, and 15 minutes, following the protocol. Various force-time metrics were extracted from the jump tasks, and linear mixed models with subject ID as the random factor, and time as the fixed factor were used to investigate changes across the three time points. To account for the performance during the repeat sprint protocol, a second, two factor model was performed with time and repeat sprint ability (RSA) as the fixed factors. Study results indicated that the sample as a whole merely experienced fatigue-induced decreases in jump height from pre-repeat sprint ability protocol (pre-RSA) within the CMJ compared to two minutes post-repeat sprint ability protocol (post-RSA_1_) and 15 minutes post-repeat sprint ability protocol (post-RSA_2_), while jump height within the DJ was only significantly different from pre-RSA at post-RSA_1_. Further, despite the implementation of the fatiguing RSA protocol, over the course of the three time-points, participants seemed to perform the two jump tasks more efficiently, seen through significantly lower contraction times, greater eccentric (ECC) peak power, and greater ECC mean deceleration force within the CMJ following the RSA task. The two-factor model revealed that several significant time*RSA interactions were found for metrics such as ECC peak velocity and peak power in the CMJ, as well as reactive strength index in the DJ. This suggests that the level of RSA influenced changes across CMJ and DJ characteristics and should be accounted for when interpreting fatigue-induced changes in neuromuscular performance.

## Introduction

Basketball is one of the most popular sports globally, and from a physical standpoint requires athletes to show proficiency in a number of motor abilities such as speed, strength, and endurance [1]. Multiple research reports have shown that during gameplay, athletes frequently perform high intensity accelerations, decelerations, jumps, as well as changes in direction [2–4]. Depending on the level of play, and the country in which an athlete is competing, schedules, especially in-season are commonly stacked with practices and games, exposing players to large internal and external workloads. For instance, one league that is known to have a very dense game-schedule, is the National Basketball Association (NBA). It has been shown that over recent years, professional athletes in the NBA experienced a rate of game-related injuries that is twice as high compared to collegiate basketball, in which the game schedule is less dense [5]. It seems apparent that workload and fatigue-monitoring strategies may be of interest to sport-science practitioners working with athletes that perform in such environments that are dense in games and practices. Given the commonality of movements in basketball that involve the use of the stretch-shortening-cycle (SCC), monitoring strategies have been implemented to gain insights into athletes’ neuromuscular performance, which may be influenced by the workloads they are exposed to. By definition, the SSC describes a phenomenon consisting of an eccentric phase or stretch followed by an isometric transitional period (amortization phase), leading into an explosive concentric action [6], and common tests to monitor performance involving the SSC are for instance that vertical countermovement jump (CMJ), or drop jump (DJ). Both of these movements involve a rapid eccentric phase, followed by an amortization phase, leading into an explosive concentric action. While still limited in existing evidence, these tests have been shown to be sensitive toward different kinds of fatigue [7,8]. For instance, Gathercole et al. [8] has proposed that assessments involving an eccentric phase (e.g., CMJ, DJ) may provide superior sensitivity with regards to neuromuscular fatigue detection. While it is common to focus on metrics such as maximal jump height from assessments such as the CMJ or DJ, when using force-platforms, a more detailed picture may be painted of the athletes jump strategy by analyzing different force-time characteristics [9]. For instance, in a different study, Gathercole et al. [10] has proposed that the CMJ may be a viable option for neuromuscular fatigue detection. The same group of authors also encouraged practitioners to analyze force-time metrics that show the specific jump strategy an athlete may use. Even in highly trained subjects, jump strategies in a seemingly simple task such as the CMJ may differ vastly among athletes from a kinetic and kinematic standpoint [11].

While it has been suggested that assessments such as the CMJ or DJ may be used with regards to neuromuscular fatigue detection, evidence is still limited. There is still little consensus as to which vertical jump metrics seem to be most sensitive towards fatigue [12]. More specifically, said studies often consist of small samples, and fail to account for the type of fatigue athletes experience within their sport, as well as an individual athlete’s performance or level of fitness during such fatiguing tasks. Previous research has established that during basketball gameplay, athletes perform up to 105 high-intensity bouts, lasting between two and six seconds, occurring every 21 seconds [13]. Therefore, it may be speculated that the ability to perform repeated sprints during a game, with minimal fatigue may be of importance to basketball athletes [14]. For instance, Abdelkrim et al. [15] suggested that during games, male junior basketball players experienced significant decreases in distances covered at high-speed running distances, and that game maximal, and high-speed running were significantly correlated with endurance performance. Beyond that, repeat sprint ability (RSA) protocols have been shown to induce acute levels of fatigue [16,17], which are comparable to exertion levels experienced in sport competition, and performance during such protocols is easily quantifiable.

With the previously highlighted thoughts in mind, the aims of this study were threefold: The primary aim was to investigate how the fatigue induced by repeated sprints acutely affects neuromuscular performance as quantified via the CMJ and DJ, within a sample of recreationally trained basketball athletes. Further, the second aim was to account for the level of participant fatigue and fitness through performance within the RSA protocol, to quantify how this may affect changes in neuromuscular performance. To quantify if a learning effect was present which might have influenced outcomes, intra-test coefficient of variation percentages for selected metrics were compared between time-points. Researchers hypothesized that following the fatiguing RSA protocol, participants would experience decreases in neuromuscular performance that would affect metrics related to task outcomes (e.g., jump height or force-related metrics), as well as task strategies (e.g., eccentric velocity, countermovement depth, contraction time).

## Materials and methods

### Experimental approach to the problem

A cross-sectional research design was utilized to investigate the fatiguing response of, as well as the performance within a sport-specific repeat sprint ability protocol on acute changes in vertical neuromuscular performance measures. Study procedures required participants to complete a single testing session. In the following order, participants’ written consent was obtained, followed by the measurement of anthropometric characteristics (e.g., height and weight). Participants performed a dynamic warm up, which was led by a Certified Strength and Conditioning Specialist. Next, participants completed pre-RSA testing, which consisted of three CMJ’s and DJ’s performed on a force plate. Following pre-RSA testing, participants performed a commonly implemented repeat sprint ability assessment. This assessment was implemented to (1) induce a level of acute fatigue, and (2), measure participants repeat sprint ability. Following the RSA protocol, participants were provided with 2 minutes of passive rest, and were then re-tested within the CMJ and DJ tasks performed during pre-RSA testing. Then, participants were given an additional 15 minutes of rest, after which pre-RSA testing tasks were re-done one last time. Participants were provided with a low-intensity, non-fatiguing dynamic warm up at the 10-minute mark and started the last testing block once the 15 minutes were up. The 2-, and 15-minute rest periods were chosen to reflect a typical basketball quarter, and a half-time break, respectively (professional rules basketball).

### Subjects

The sample for this investigation consisted of 25 recreationally trained basketball athletes (n = 23 male, n = 4 female, age = 21.4 ± 2.5 years, height = 182.7 ± 8.3 cm, weight = 77.4 ± 9.91 kg), with at least four years of recent, organized basketball playing experience (e.g., High School, Club, College, etc.) within the United States of America. All participants reported no acute injury or illness prior to participation. All testing procedures were approved by the Universities Institutional Review Board, and all subjects provided written consent prior to the commencement of data collection (STUDY00149304). Subjects were recruited between October and December of 2022, and only the principal investigator of the study had access to information that could identify individual participants during or after data collection. For further analyses, all data were de-identified. All data was collected by the principal investigator of the study, and subjects were instructed to refrain from intense physical exercise the 24 hours leading up the data collection. All subjects wore comfortable athletic clothing, and basketball-specific shoes.

### Warm up and familiarization

Participants performed a dynamic warm up consisting of a number of dynamic stretches and exercises such as lateral lunges, forward and backward jogging, high knees, as well as horizontal and vertical skips and bounds. Further, to familiarize participants with the assessments, three practice repetitions within the CMJ, and DJ were completed, respectively, following a detailed instruction about each task, by the principal investigator.

### Countermovement jump testing

Athletes performed a total of three CMJs. To minimize the effect of acute jump fatigue on jump performance, each jump was separated by a 15-30-second rest interval. Data were recorded using portable dual force plates (ForceDecks Max, Vald Performance Pty Ltd., Brisbane, Australia), sampling at 1000 Hz. The force plates were zeroed prior to each subject’s test. Athletes were given instructions to step onto the force plate and to stand as still as possible for 2–3 seconds, and then to jump as fast and as high as possible while keeping their hands on their hips during the entire movement. Verbal encouragement was provided to ensure maximal effort was given for each jump. CMJ metrics of interest, and metric definition can be found in Table 1. The eccentric phase was defined as the phase containing negative velocity, with the deceleration phase being a subphase of the eccentric phase, starting at peak eccentric velocity, and ending with the conclusion of the eccentric phase. The concentric phase began immediately following the eccentric phase at zero center of mass velocity and ended at take-off. Further specifications of different phases during the CMJ can be found elsewhere [18,19]. In line with previous research, kinetic CMJ metrics from the eccentric and concentric phase of the jump were selected, in addition to performance metrics, and metrics that reflect the jump strategy used [20]. For simplicity, force-time metrics were classified as kinetic metrics (KIN), strategy metrics (STRAT), and ratio metrics (RATIO), as well as performance metrics (PERF). At pre-RSA, coefficient of variation (CV%) for all CMJ metrics ranged from 1.1% to 5.6% percent.

**Table 1:** Countermovement jump force-time metric definitions and classifications.

**Countermovement Jump Metrics**	**Definition (Metric Classification)**
ECC Mean Force (N)	Average force generated during the ECC phase (KIN)
ECC Peak Power (W)	Peak power generated during the ECC phase of the jump (KIN)
ECC Peak Velocity (m/s)	Maximal velocity obtained during the ECC phase (STRAT)
ECC:CON Mean Force Ratio (%)	Ratio of mean forces in the ECC and CON phases (STRAT)
ECC Deceleration RFD (N•s^-1^)	The average change in force over time during the ECC deceleration phase (KIN)
Contraction Time (ms)	Duration from start of the countermovement until take-off (STRAT)
Countermovement Depth (cm)	Lowest center of mass displacement, transition from ECC to CON phase (STRAT)
CON Mean Force (N)	Average force generated during the CON phase (KIN)
Force at Zero Velocity (N)	Total force at the instance velocity is zero prior to take-off (KIN)
Jump Height (cm)	Maximal jump height via impulse—momentum calculation (PERF)
Peak Power (W)	Peak power generated during the jump (PERF)
Positive Impulse (N•s)	Impulse observed above system weight (KIN)
RSI-modified (Ratio)	Jump height divided by contraction time (RATIO)
**Drop Jump Metrics**	**Definition (Metric Classification)**
Contact Time (s)	Time spent in contact with the ground between landing and take-off (STRAT)
Jump Height (cm)	Maximal jump height via impulse—momentum calculation (PERF)
RSI (Ratio)	Jump height divided by contact time (RATIO)

Note: ECC = eccentric; CON = concentric; RSI = reactive strength index, TRAD = Traditional, STRAT = Strategy, MIX = Mixture of Traditional and Strategy.

### Drop jump testing

Following the performance of three CMJs, participants completed three bilateral DJ off a 30-centimeter box. Again, to minimize the effect of acute fatigue on jump performance, each jump was separated by a 15-30-second rest interval. Athletes were given instructions to step onto the 30 cm box placed directly behind the force plates, and to then drop off the box, land with two feet, to minimize the time in contact with the ground while still aiming to jump as high as possible, while keeping their hands on their hips during the entire movement. Verbal encouragement was provided to ensure maximal effort was given for each jump. DJ metrics of interest, and metric definition can be found in Table 1. For the DJ metrics, CV% ranged from 6.6% to 9.0%.

### Repeat sprint ability assessment

Procedures for the RSA assessment were adapted from Castagna et al. [16], given the sport specific nature of the tasks. Following block one of horizontal and vertical deceleration testing, participants performed the RSA assessment, which consisted of 10, 30-meter shuttle sprints (15m + 15m), separated by 30 seconds of passive recovery. This RSA protocol has been shown to acutely induce substantial levels of fatigue within similar populations, as measured via blood lactate concentrations [16,17]. Participants were instructed to initiate each shuttle sprint from a two point/staggered stance, with their front foot placed on a taped line, 30 cm’s behind the set of timing gates used to measure completion times of the shuttle sprints (Brower Timing Systems, Draper, UT). Participants sprinted linearly over 15 meters, planted their foot on the 15-meter marker, performed a 180-degree turn, and sprinted back through the start line as fast as possible. A member of the research team ensured athletes made contact with the 15-meter marker for each run. Participants were instructed to sprint through the finish line, to decelerate, and walk back to the starting line, to await the initiation of the next run. To avoid pacing, participants were strongly encouraged to complete each shuttle sprint as fast as possible. To assess performance and fatigue during the RSA assessment, percentage sprint performance maintained (%-maintained) was used, given its higher level of reliability compared to the traditional fatigue index (FI) used during RSA assessments [21]. %-maintained was calculated using the following equation: “%-maintained = (best sprint time / mean sprint time) *100”.

### Statistical analyses

Normality of residuals was assessed using Q-Q plots and residuals histograms. To investigate changes in CMJ and DJ metrics across the three time-points (i.e., pre-RSA, post-RSA_1_, post-RSA_2_), a linear mixed effect model was used, with participant ID as the random factor, and time as the fixed factor. In case of a univariate effect, further pairwise t-test comparisons were performed using a Bonferroni correction. Further, a two-factor linear mixed model was deployed investigating changes across the three timepoints, taking into consideration participant performance during the RSA protocol as a proxy measure of fatigue and fitness. To do so, the sample was divided into two groups based on a median split analyses for %-maintained, and another linear mixed effect model was run with participant ID as the random factor, and time, as well as group (RSA-classification) as the fixed factors. In the case of significant interaction effects (time*RSA-classification) further pairwise t-tests using a Bonferroni correction were performed. In case of significant differences between time-points, Cohen’s d effect sizes were calculated to represent the magnitude of difference, and were interpreted as negligible (≤0.10), small (0.11–0.50), moderate (0.51–0.75), and large (>0.75) [22]. Lastly, a third, supplementary analysis was performed with participant ID as the random factor, time as the fixed factor, and the CV% for each respective metric at the three time points as the dependent variable. This was done to understand if a learning effect was present that might have influenced fatigue-induced changes in force-time characteristics. Given the exploratory nature of this study in exploring potential links between selected force-time metrics and fatigue, in conjunction with the low risks associated with making type one errors in our investigation, results were interpreted at the 0.10 alpha level. To determine the required sample size, an a priori G*Power-based power analysis (G*Power version 3.1) was conducted with an alpha level of 0.10, a desired power of 0.80, and an effect size_f_ of 0.40 (ANOVA: Repeated measures, within-between interaction). The power analysis suggested that a minimum of 10 subjects per group (RSA-classification), and 20 subjects total would be required to achieve the desired power. All other analyses were performed within the RStudio Software (Version 1.4.1106).

## Results

Looking at the CMJ metrics, significant univariate effects for time were found for jump height (F = 12.2, p < 0.001), with participants showing significantly lower jump heights at post-RSA_1_ and post-RSA_2_, compared to pre-RSA. Further, significant univariate effects for time were found of countermovement depth (F = 11.5, p < 0.001), with participants performing a significantly shallower countermovement at post-RSA_1_ and post-RSA_2_, compared to pre-RSA. On the other hand, significant univariate effects for time were found for contraction time (F = 11.2, p < 0.001), with participants showing significantly shorter contractions times at post-RSA_1_ and post-RSA_2_, compared to pre-RSA. Similar findings were found for eccentric and concentric duration. Additional significant univariate effects for time were found for ECC mean braking force (F = 3.47, p = 0.039), as well as ECC mean deceleration force (F = 3.38, p = 0.041), with participants generating significantly greater magnitudes of force at post-RSA_2_ compared to pre-RSA. Lastly, significant univariate effects for time were also found for concentric mean force (F = 4.35, p = 0.018), as well as eccentric peak power (F = 3.60, p = 0.035) with participants generating significantly greater magnitudes of concentric mean force at post-RSA_2_ compared to post-RSA_1_, and significantly greater eccentric peak power at post-RSA_2_ compared to pre-RSA.

**Fig 1:**
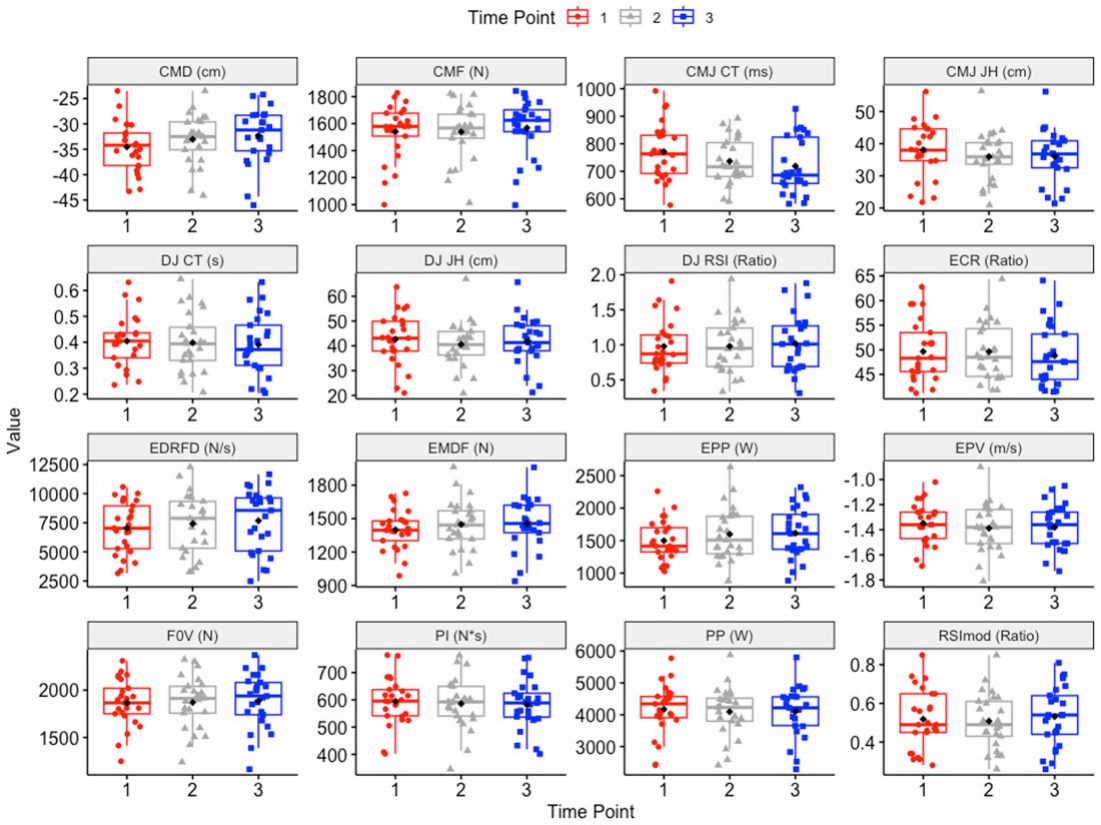
Boxplots with data distribution, mean, median, and first and third quartiles, visualizing changes in all CMJ and DJ metrics across the three time-points analyzed. *Note: Time-point 1 = pre-rsa, time-point 2 = post-rsa_1_, time-point 3 = post-rsa_2_. CMD = concentric mean duration, CMF = concentric mean force, CMJ CT = CMJ contraction time, CMJ JH = CMJ jump height, DJ CT = DJ contact time, DJ JH = DJ jump height, DJ RSI = DJ reactive strength index, ECR = ECC:CON mean force ratio, EDRFD = ECC Deceleration RFD, EMDF = ECC mean deceleration force, EPF = ECC peak force, EPP = ECC peak power, EPV = ECC peak velocity, F0V = force at zero velocity, PP = peak power, RSImod = reactive strength index modified, PI = positive impulse.

With regards to the DJ, only jump height showed a significant univariate effect (F = 2.76, p = 0.073), with participants performing significantly lower jump heights at post-RSA_1_ compared to pre-RSA. Table 2 shows raw data for all metrics of interest, across all three time-points, while Fig 1 visually depicts data for all metrics of interest, across all three timepoints.

**Table 2:** Descriptive statistics for CMJ and DJ force-time metrics across the three time-points.

Countermovement Jump Metrics	Pre-RSA	Post-RSA_1_	Post-RSA_2_
ECC Mean Deceleration Force (N)	1401 ± 176	1448 ± 219	**1454 ± 225** *
ECC Peak Velocity (m/s)	-1.35 ± 0.16	-1.39 ± 0.19	-1.38 ± 0.18
ECC Peak Power (W)	1499 ± 310	1598 ± 407	**1615 ± 391** *
ECC:CON Mean Force Ratio (Ratio)	49.7 ± 6.15	49.6 ± 5.80	**47.6 ± 6.30** *
ECC Deceleration RFD (N•s^-1^)	7034 ± 2216	7419 ± 2586	7674 ± 2696
Contraction Time (ms)	772 ± 102	**737 ± 82.9** ‡	**719 ± 99.8** ^†^
Countermovement Depth (cm)	-34.5 ± 4.84	**-33.0 ± 4.90** ^†^	**-31.2 ± 5.39** ‡
CON Mean Force (N)	1541 ± 202	1539 ± 203	1568 ± 213
Force at Zero Velocity (N)	1865 ± 251	1870 ± 276	1886 ± 287
Jump Height (cm)	38.1 ± 8.50	**35.9 ± 7.62** ^†^	**36.8 ± 8.15** ‡
Peak Power (W)	4170 ± 798	4094 ± 772	4106 ± 791
Positive Impulse (N•s)	594 ± 87.8	586 ± 94.2	582 ± 91.3
RSI-modified (Ratio)	0.52 ± 0.16	0.51 ± 0.14	0.53 ± 0.16
**Drop Jump Metrics**			
Contact Time (s)	0.41 ± 0.10	0.40 ± 0.11	0.39 ± 0.12
Jump Height (cm)	42.7 ± 10.3	**40.6 ± 9.16** *	41.8 ± 9.58
RSI (Ratio)	0.98 ± 0.38	0.98 ± 0.38	1.02 ± 0.41

Note: Bold = significantly different from baseline.

* = small effect size.

^†^ = moderate effect size.

^‡^ = large effect size.

Within the CMJ, two factor results revealed significant time*fatigue-class interactions for peak power (F = 3.32, p = 0.045). Further Bonferroni corrected pairwise comparisons suggested significant decreases in peak power within the high fatigue-class group between pre-RSA and post-RSA_1_ (p = 0.050), which were not reflected in the low fatigue-class group. Additionally, significant time*fatigue class interaction for ECC peak velocity were observed (F = 2.62, p = 0.084). Pairwise t-tests suggested significant increases in ECC peak velocity from pre-RSA to post-RSA_1_ for the low fatigue class group (p = 0.096), while the high fatigue class group experienced slight, non-significant decreases in ECC peak velocity across the three time points. Lastly, significant time*RSA class interactions were observed for positive impulse (F = 4.17, p = 0.022), with the low RSA group experiencing significant decreases in impulse between pre-RSA and post-RSA_2_ (p = 0.027), while the high RSA group experienced non-significant improvements.

**Fig 2:**
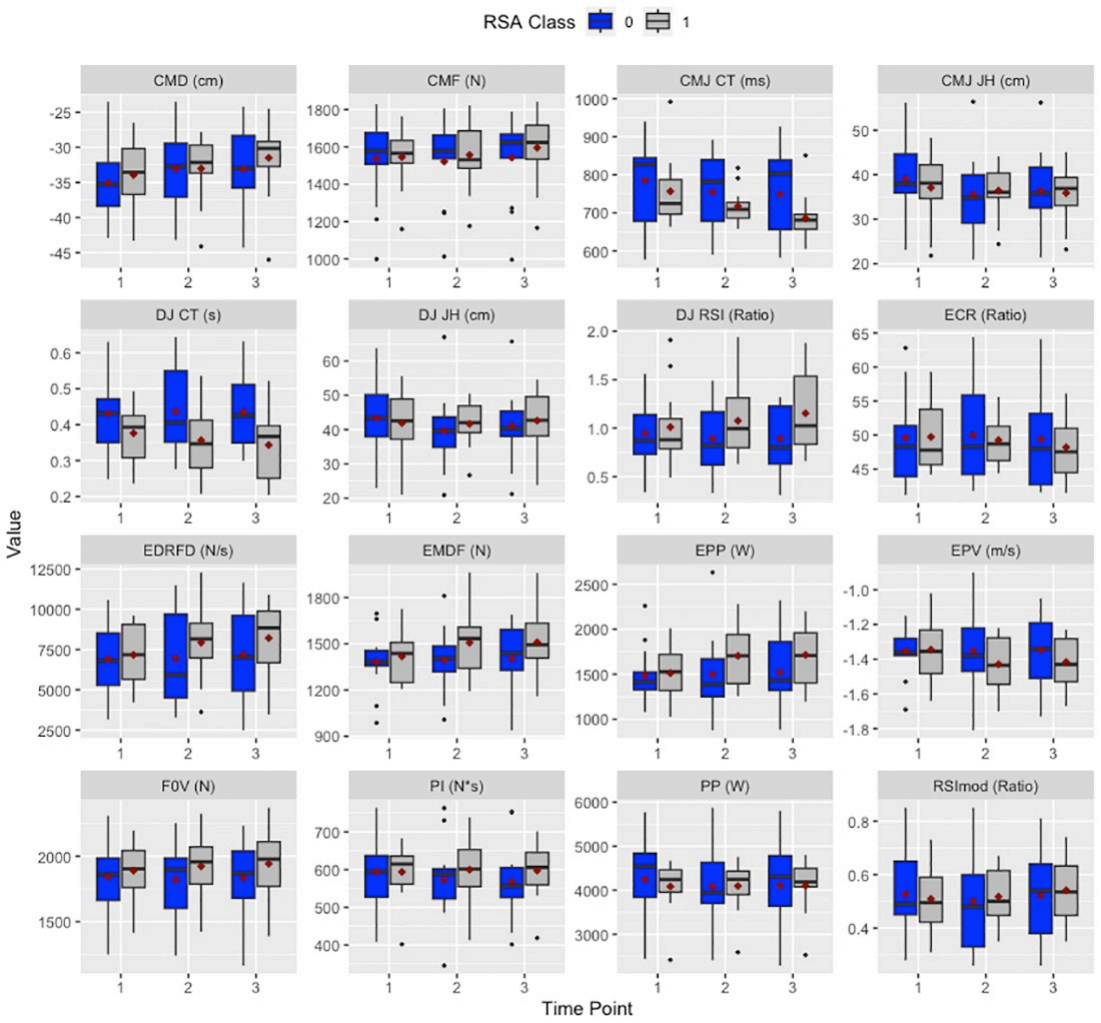
Boxplots with mean median and first and third quartiles visualizing changes in all CMJ and DJ metrics across the three time-points, broken down by RSA classification. *Note: Time-point 1 = pre-rsa, time-point 2 = post-rsa_1_, time-point 3 = post-rsa_2_. RSA class 1 = high RSA performance, RSA class 2 = low RSA performance, CMD = concentric mean duration, CMF = concentric mean force, CMJ CT = CMJ contraction time, CMJ JH = CMJ jump height, DJ CT = DJ contact time, DJ JH = DJ jump height, DJ RSI = DJ reactive strength index, ECR = ECC:CON mean force ratio, EDRFD = ECC Deceleration RFD, EMDF = ECC mean deceleration force, EPF = ECC peak force, EPP = ECC peak power, EPV = ECC peak velocity, F0V = force at zero velocity, PP = peak power, RSImod = reactive strength index modified.

Similarly, within the DJ, two factor results revealed significant time*fatigue-class interactions for RSI (F = 3.81, p = 0.029). Further Bonferroni corrected pairwise comparisons suggested non-significant increases in RSI between pre-RSA and post-RSA_2_ for the low fatigue-class group (p = 0.134), while the high fatigue-class group experienced non-significant decreases in RSI from pre-RSA to post-RSA_1_ and post-RSA_2_. Table 3 shows raw data for the two-factor analysis, while Fig 2 visually depicts data from the two-factor analysis, for all metrics of interest.

**Table 3:** Descriptive statistics for CMJ and DJ force-time metrics across the three time-points and by RSA-classification.

Countermovement Jump Metrics	RSA-Class	Pre-RSA	Post-RSA_1_	Post-RSA_2_
ECC Mean Deceleration Force (N)	High	1418 ± 165	1403 ± 221	1510 ± 221
	Low	1384 ± 191	1502 ± 211	1403 ± 224
ECC Peak Velocity (m/s)	High	-1.34 ± 0.19	**-1.43 ± 0.16** ‡	-1.42 ± 0.15
	Low	-1.35 ± 0.14	-1.35 ± 0.22	-1.35 ± 0.20
ECC Peak Power (W)	High	1512 ± 311	**1705 ± 353** ‡	**1715 ± 335** ‡
	Low	1486 ± 322	1499 ± 442	1522 ± 429
ECC:CON Mean Force Ratio (Ratio)	High	49.7 ± 5.13	49.3 ± 4.02	**48.2 ± 4.86** ‡
	Low	49.9 ± 7.18	50.0 ± 7.22	49.4 ± 7.54
ECC Deceleration RFD (N•s^-1^)	High	7166 ± 1934	7931 ± 2251	8212 ± 2408
	Low	6913 ± 2522	6947 ± 2868	7177 ± 2944
Contraction Time (ms)	High	757 ± 91.1	717 ± 47.0	**686 ± 64.0** ‡
	Low	785 ± 114	754 ± 105	749 ± 119
Countermovement Depth (cm)	High	-33.9 ± 4.74	-33.0 ± 4.61	**-31.5 ± 5.60** ‡
	Low	-35.1 ± 5.07	**-33.1 ± 5.34** ^†^	**-33.1 ± 5.30** ‡
CON Mean Force (N)	High	1545 ± 164	1557 ± 189	1595 ± 197
	Low	1537 ± 239	1522 ± 221	1542 ± 231
Force at Zero Velocity (N)	High	1890 ± 217	1923 ± 265	1943 ± 274
	Low	1842 ± 285	1821 ± 287	1833 ± 299
Jump Height (cm)	High	37.1 ± 7.87	36.4 ± 5.92	35.9 ± 6.66
	Low	39.1 ± 9.26	**35.6 ± 9.15** ‡	**36.4 ± 9.60** ‡
Peak Power (W)	High	4086 ± 598	4099 ± 594	4104 ± 603
	Low	4248 ± 965	**4090 ± 932** ^†^	4108 ± 957
Positive Impulse (N•s)	High	594 ± 76.2	600 ± 83.3	598 ± 76.5
	Low	595 ± 101	573 ± 105	**568 ± 104** ^†^
RSI-modified (Ratio)	High	0.51 ± 0.14	0.52 ± 0.10	0.54 ± 0.13
	Low	0.53 ± 0.17	0.50 ± 0.18	0.52 ± 0.18
**Drop Jump Metrics**				
Contact Time (s)	High	0.38 ± 0.08	0.36 ± 0.11	0.34 ± 0.11
	Low	0.43 ± 0.11	0.44 ± 0.11	0.44 ± 0.11
Jump Height (cm)	High	41.9 ± 9.9	41.6 ± 6.7	42.6 ± 8.4
	Low	43.4 ± 11.0	**39.6 ± 11.2** ^†^	41.1 ± 10.8
RSI (Ratio)	High	1.01 ± 0.41	1.08 ± 0.38	1.15 ± 0.44
	Low	0.95 ± 0.36	0.89 ± 0.37	0.89 ± 0.35

Note: Bold = significantly different from baseline.

* = small effect size.

^†^ = moderate effect size.

^‡^ = large effect size.

**Fig 3:**
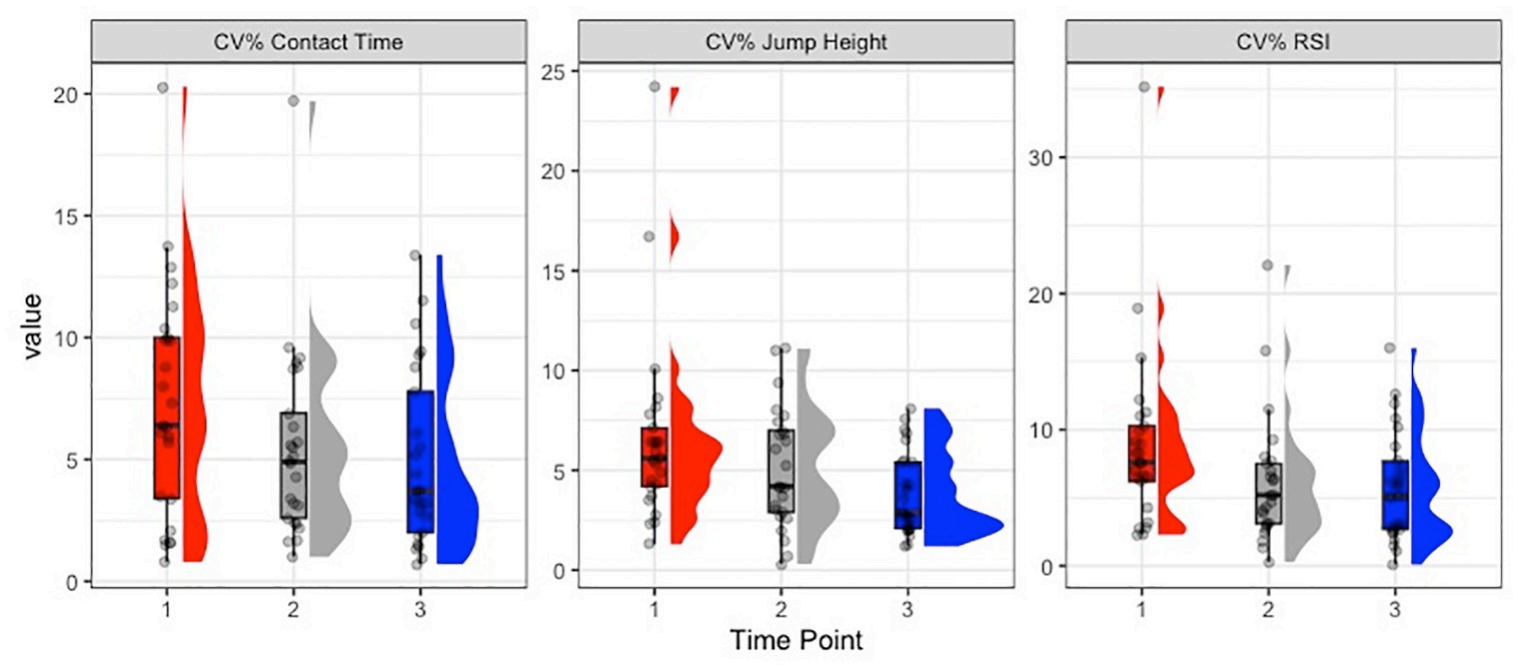
Raincloud plots with median, quartile, density and individual data points for CV%’s of the DJ metrics across the three time-points analyzed. *Note: *Time-point 1 = pre-rsa*, *time-point 2 = post-rsa*_*1*_, *time-point 3 = post-rsa*_*2*._

Examination of how within trial CV%’s changed across the three time-points revealed that CV%’s for CMJ metrics remained unchanged from a statistical standpoint. On the other hand, significant univariate effects for time were found for jump height CV%’s within the DJ task (F = 4.87, p = 0.012), with CV%’s being significantly lower at post-RSA_2_ compared to pre-RSA. Further univariate effects for time were found for contact time CV%’s (F = 2.60, p = 0.084), as well as RSI CV%’s (F = 2.74, p = 0.071), indicating that CV%’s decreased from pre-RSA to post-RSA_1_, and post-RSA_2_. Fig 3 visualizes said decreases in CV%’s, through the linearly decreasing spread of the data.

## Discussion

The primary aim of the present study was to investigate how the fatigue induced through a repeat sprint protocol acutely affected different measures of neuromuscular performance. Different force-time metrics from the CMJ and DJ task performed prior to the RSA protocol, as well as 2 minutes, and 15 minutes following the RSA protocol were analyzed. Our data suggested that from a statistical standpoint, the study sample as a whole merely experienced fatigue-induced decreases from pre-RSA in jump height within the CMJ compared to post-RSA_1_ and post-RSA_2_, while jump height within the DJ was only significantly different from pre-RSA at post-RSA_1_. Further, despite the implementation of the fatiguing RSA protocol, over the course of the three time-points, participants seemed to perform the two jump tasks more efficiently, seen through significantly lower contraction times, greater ECC peak power, and greater ECC mean deceleration force within the CMJ. In addition, a significantly shallower countermovement depth was observed at post-RSA_1_ and post-RSA_2_, compared to pre-RSA. While a shallower countermovement is likely related to a faster contraction time, other research within highly trained athletes has shown that a deeper and faster countermovement was related to greater jump heights [23]. In our study, participants performed a shallower countermovement and achieved lower jump heights following the fatiguing RSA protocol. It may be speculated that a lower countermovement depth is a fatigue-induced response and may be due in part to altered lower limb stiffness. Additionally, our data may propose that in the presence of a shorter contraction time, participants achieved a lower positive impulse, which helps explain the depressed jump heights. Further, Merrigan et al. [23] suggested that lower ECC:CON force ratios were related to greater jump performance. In our study, participants showed significantly lower ECC:CON force rations at post-RSA_2_ compared to pre-RSA, suggesting a more optimal jump strategy that was not accompanied by greater jump heights, likely due to the fatigue of the RSA protocol. These results are in line with previous findings reporting depressed performance in metrics such as flight time, and relative net impulse immediately following fatiguing bouts of exercise, while strategy-based metrics such as flight-time:contraction-time, or time to peak power showed more notable changes at later time-points (e.g., 24–72 hours following fatiguing exercise) [7,8]. The previous findings alone show the multifaceted nature of analyzing different CMJ metrics, and the notion that simply focusing on one metric likely is somewhat shortsighted.

Within our sample, it is reasonable to suggest that the level of fitness and therefore the level of fatigue experienced throughout and following the RSA protocol differed between participants. As mentioned within the introduction, few studies investigating the effects of fatigue on neuromuscular performance account for the performance of the participants during the fatiguing task and operate under the assumption that fatigue is the same for everyone in the sample. Therefore, a secondary aim of this study was to investigate the effect of a fatiguing RSA protocol on CMJ and DJ force-time metrics, also accounting for the participants performance during the RSA protocol.

Results from the two-factor model revealed that only focusing on the group mean may not fully reflect how participants respond to a fatiguing RSA protocol from a neuromuscular standpoint. For instance, significant time*RSA classification effects were observed for ECC peak velocity, with those participants with superior RSA experiencing significant increases in ECC peak velocity, and those with inferior RSA experienced slight non-significant decreases. While speculative, this may suggest that the fatigue of the RSA protocol hindered the participants of the low RSA group in employing a faster eccentric contraction, as seen within the high RSA group. Moreover, with regards to peak power, participants with lower RSA experienced significant decreases in from pre-RSA to post-RSA_1_, while those with higher RSA experienced slight, non-significant increases in peak power following the fatiguing task. Similarly, a significant time*RSA classification interaction was observed for RSI in the DJ. While Bonferroni-corrected pairwise comparisons revealed no further statistical significance, participants with greater RSA experienced increases in RSI following the fatiguing protocol, while those with lesser RSA experienced decreases. Lastly, significant time*RSA class interactions were observed for positive impulse, with the low RSA group experiencing significantly decreases in impulse between pre-RSA and post-RSA_2_, while the high RSA group experienced non-significant improvements. These findings highlight the potential need to quantify athlete fitness when studying the effects of fatigue on neuromuscular performance.

As part of this study, different ratio metrics were analyzed, such as RSImod and RSI. These are generally composed of a traditional kinetic metric and a strategy-based metric. Previous research has indicated the importance of analyzing the individual component of ratio metrics, given that a change in performance could result from an increase or decrease in either of the two metrics, as well as the fact that ratio metrics tend to be noisier [24]. These suggestions are further reflected within our results. For instance, when looking at the DJ, while different in the magnitude of change, both the high and low RSA group experienced decreases in jump height from pre-RSA to post-RSA_1_, while contact time from pre-RSA to post-RSA_1_ decreased for the high RSA group, and slightly increased for the low RSA group, suggesting that the group with superior RSA was able to present with a likely more efficient jump strategy, even following the fatiguing protocol.

One factor that requires attention when interpreting the results of our study is the task familiarity of our sample, with regards to performing CMJ’s and DJ’s, especially while maintaining their hands on the hips throughout the entire movement. The majority of the participants had no prior, habitual exposure to either task, suggesting that even though clear instructions as well as three practice jumps for each task were provided during the warm-up, a learning effect might have still been present across the three time-points, which might’ve influenced the results. As shown by our data, an example of this potential learning effect is the significant decrease in within trial CV%’s for the three DJ metrics that was observed across the three time-points (Fig 3). This learning effect should be accounted for, especially when introducing neuromuscular assessments to populations with limited previous experience.

Previous studies investigating the usefulness of the CMJ and DJ to detect neuromuscular fatigue have suggested that it is of importance to consider the jump strategy used when interpreting results [10], and others have proposed the thought that traditional performance metrics such as jump height or peak power may be more useful with regards to performance profiling and tracking, rather than fatigue identification [25,26]. Interestingly, in our study, when considering the whole sample, the only metrics that significantly decreased, following the RSA-protocol, were CMJ jump height, DJ jump height, and countermovement depth, while strategy metrics such as contraction time and ECC:CON mean force ratio improved, possibly attributed to a learning effect. Therefore, while somewhat speculative, we suggest that practitioners closely monitor strategy-based metrics when they are confident that athletes are familiar with the movement, and consistently present with similar jump strategies in non-fatigued conditions. Further, it seems that when monitoring populations with less previous exposure to neuromuscular performance assessments, outcome-based metrics such as jump height or peak power might be more suitable when interested in analyzing metrics in response to fatigue or fluctuating workloads.

Limitations and potential directions of future research should also be taken into consideration when interpreting the results of our study. As mentioned within the previous paragraph, to account for the learning effect associated with measures of neuromuscular performance, familiarization periods should be implemented. Further, CMJ and DJ performance was only re-assessed 2 minutes and 15 minutes post-RSA, making it difficult to conclude whether or not impairments in neuromuscular function remained present at later time-points. Future research may aim to replicate methods with highly trained athletes over extended periods of time, such as over the course of a season, accounting for the fatigue that comes with participation in training and competition. Lastly, readers should be cognizant that positive changes from pre-RSA to post-RSA_1_ could have been influenced by a potentiation effect, especially within the group possessing greater RSA, given that potentiation effects have been reported to not dissipate until 5–6 minutes following exercise [27].

## Conclusion

Practitioners may find findings from this study insightful when choosing neuromuscular performance tests and metrics to gain insights into athlete’s readiness and fatigue levels. It is common for high-level athletes to perform multiple training sessions in one day, as well as on back-to-back days. Further, it is not uncommon for practitioners to implement assessments of neuromuscular function prior to and following a game or competition. Performing neuromuscular performance assessments such as the ones used in this study, following the commencement of a training session or competition, may provide insights into how well athletes responded to the workload they were exposed to, and whether or not additional recovery options may be considered prior to the next training session or competition, in order to enhance readiness and performance. Metrics such as jump height or peak power may be sensitive to acute fatigue in populations with little experience with neuromuscular assessments such as the CMJ or DJ, while strategy metrics should also be considered in populations that present with sufficient test familiarity.

## Supporting information

S1 File (XLSX)
